# The Apolipoprotein M–Sphingosine-1-Phosphate Axis: Biological Relevance in Lipoprotein Metabolism, Lipid Disorders and Atherosclerosis

**DOI:** 10.3390/ijms14034419

**Published:** 2013-02-25

**Authors:** Bas W. C. Arkensteijn, Jimmy F. P. Berbée, Patrick C. N. Rensen, Lars B. Nielsen, Christina Christoffersen

**Affiliations:** 1 Department of Clinical Biochemistry, Rigshospitalet, Blegdamsvej 9, 2100 Copenhagen, Denmark; E-Mails: bas.arkensteijn@gmail.com (B.W.C.A.); larsbo@rh.dk (L.B.N.); 2 Department of Biomedical Sciences, University of Copenhagen, Copenhagen 2200, Denmark; 3 Department of Endocrinology and Metabolic Diseases, Leiden University Medical Center, Leiden 2300, The Netherlands; E-Mails: J.F.P.Berbee@lumc.nl (J.F.P.B.); P.C.N.Rensen@lumc.nl (P.C.N.R.); 4 Pharmacology Unit, Department of Medicine, University of Patras Medical School, Rio TK. 26500, Greece; 5 Department of Clinical Medicine, University of Copenhagen, Copenhagen 2200, Denmark

**Keywords:** apolipoprotein M, sphingosine-1-phosphate, atherosclerosis, lipoprotein metabolism

## Abstract

Apolipoprotein M (apoM) is a plasma apolipoprotein that mainly associates with high-density lipoproteins. Hence, most studies on apoM so far have investigated its effect on and association with lipid metabolism and atherosclerosis. The insight into apoM biology recently took a major turn. ApoM was identified as a carrier of the bioactive lipid sphingosine-1-phosphate (S1P). S1P activates five different G-protein-coupled receptors, known as the S1P-receptors 1–5 and, hence, affects a wide range of biological processes, such as lymphocyte trafficking, angiogenesis, wound repair and even virus suppression and cancer. The ability of apoM to bind S1P is due to a lipophilic binding pocket within the lipocalin structure of the apoM molecule. Mice overexpressing apoM have increased plasma S1P concentrations, whereas apoM-deficient mice have decreased S1P levels. ApoM-S1P is able to activate the S1P-receptor-1, affecting the function of endothelial cells, and apoM-deficient mice display impaired endothelial permeability in the lung. This review will focus on the putative biological roles of the new apoM–S1P axis in relation to lipoprotein metabolism, lipid disorders and atherosclerosis.

## 1. Introduction

High density lipoprotein (HDL)-cholesterol correlates inversely with cardiovascular risk. Therefore, focus on developing drugs targeting HDL metabolism is high. HDL is a main effector in reverse cholesterol transport, where it mediates transfer of cholesterol from peripheral cells, likely including those in the vessel wall, to the liver for excretion [1]. HDL contains up to 75 different proteins [2]. Apolipoprotein (apo) A-I is the major protein component [2], but other apolipoproteins, like apoA-II, apoA-IV, apoA-V, apoCs, apoD, apoE and apoM, are also associated to HDL [3]. ApoA-I is produced in the liver [4] and intestine [5] and is released as lipid-poor A-I. Subsequent interaction with ABCA1 enriches the lipid-poor HDL with cellular cholesterol. HDL is modified by several enzymes before returning its cholesteryl esters to the liver for secretion of cholesterol into the bile, including lecithin: cholesterol acyltransferase (LCAT), phospholipid transfer protein (PLTP), cholesteryl ester transfer protein (CETP), endothelial lipase (EL) and hepatic lipase (HL). Apart from reverse cholesterol transport, HDL has several other functions due to the multitude of HDL-associated apolipoproteins. HDL can be anti-oxidative (due to, for example, PON1 [6]), inhibit platelet activation [7], anti-infectious (for instance, due to apoL [8] and apoC-I [9]), anti-inflammatory (due to, for example, apoA-I [10] and apoE [11]) and promote vasodilatation (by increasing NO production in endothelial cells [12]).

ApoM was first described by Xu and Dahlbäck [13] in 1999. Human apoM is a 23 or 26 kDa apolipoprotein, depending on its glycosylation status, and is mainly associated to HDL (96% is bound to HDL [14]), but also binds to low density lipoprotein (LDL), very low density lipoprotein (VLDL) and chylomicrons [13].

ApoM is a member of the lipocalin protein superfamily [15,16]. The lipocalin structure is characterized by harboring an internal binding pocket for small lipophilic molecules. Other examples of lipocalins are the mouse major urinary protein (MUP) [17] and human retinol binding protein (RBP) [18]. The apoM crystal structure also shows a hydrophobic binding pocket [15,16,19], which enables binding of fatty acids, such as myristic acid [16], and the small bioactive lipid, sphingosine-1-phosphate (S1P), identified as an endogenous ligand for apoM [19].

The apoM gene is expressed in both liver and kidney [20]. The plasma concentration of apoM is ~0.9 μmol/L [21], which is comparable to that of apoE [22]. Hence, ~5% of the HDL particles in plasma contain an apoM molecule [14]. Overexpression of apoM in mice increases plasma HDL-cholesterol, and apoM-deficiency decreases HDL-cholesterol [23]. Moreover, apoM affects the “quality” of HDL by improving potential anti-atherogenic properties of HDL [23–25]. Hence, apoM promotes the formation of pre-β HDL particles [23,24,26,27], and apoM-enrichment of HDL augments the ability of HDL to mobilize cholesterol from macrophage-derived foam cells [14,23]. ApoM-enrichment of HDL also protects against lipoprotein oxidation, possibly by scavenging oxidized phospholipids in apoM’s lipophilic binding pocket [14,23,28].

S1P was discovered by Stoffel *et al.*[29] in 1970 and first quantified by Zhang *et al.*[30] in 1991. S1P is involved in diverse biological and pathological processes, such as embryonic vascular maturation [31], lymphocyte trafficking [32] and either stimulation [33] or inhibition [34] of angiogenesis. S1P mediates these biological effects through activation of either of the five members of the S1P receptors, S1P_r1_–S1P_r5_[35], which are G-protein-coupled receptors inducing production of intracellular mediators. S1P is a C18-long fatty acid with a phosphate head group and is produced by different cell types, including erythrocytes [32], endothelial cells [36] and activated platelets [33]. The plasma concentration of S1P is ~0.5 μmol/L in humans [37]. The majority of plasma S1P (~60%) is bound to HDL, whereas ~30% is bound to albumin and a minor fraction to VLDL and LDL [38,39]. Others have seen that the amount of total S1P bound to HDL is as high as 90% [37]. S1P bound to HDL plays an important role in the ability of HDL to modulate endothelial cell function [40,41] and vasodilatation [42] through S1P_r1_ and S1P_r3_. Until recently, it was unknown how S1P binds to HDL. With the discovery of apoM as a carrier of S1P on HDL particle, new links between lipoproteins, endothelial function and atherosclerosis can be explored, and this will be the topic of the remaining part of this review.

## 2. Function of An apoM/S1P Axis

### 2.1. ApoM and Interaction with S1P and Other Ligands

The 188 amino acids of apoM include six cysteine residues that form three intramolecular disulfide bridges [13]. Human apoM has eight-stranded anti-parallel β-sheets flanked by an exterior α-helix, typical for proteins belonging to the lipocalin superfamily [13]. Hence, apoM has homology to lipocalins, such as apoD and the murine urinary protein (MUP) [15]. The crystal structure of apoM was determined using human recombinant apoM produced in *E. coli*[16]. The crystallization studies showed that the binding pocket of human apoM can complex with fatty acids like myristic acid (C14:0), palmitic acid (C16:0) and stearic acid (C18:0) [16]. Additional *in vitro* binding studies showed that S1P containing a C18-long fatty acid side chain also binds to human apoM with an IC_50_ = 0.9 μmol/L [16]. Mouse apoM that has a sequence homology with human apoM of 79% and binds S1P with similar affinity (IC_50_ = 0.95 μmol/L) [19].

To further elucidate the putative relationship between apoM and S1P, the complex of S1P and human recombinant apoM was crystallized [19]. The phosphate head group of S1P specifically interacts with two arginines (Arg98 and Arg116) and a tyrosine (Tyr100) at the entrance of the binding pocket. The amino group of S1P is bound to a glutamate (Glu136), a tyrosine (Tyr102) and an arginine (Arg143) via hydrogen bridges. The apolar tail of S1P is orientated to the inside of the binding pocket [19], explaining why fatty acids also can bind to apoM.

Besides S1P, retinoic acid has been suggested as a potential ligand of apoM [43]. Normally, more than 95% of retinoic acid binds to retinol binding protein in plasma with a Kd of ~0.1 μM [43], whereas less than 5% is bound to lipoproteins [44]. Plasma retinoic acid concentration varies between ~0.1–17 nM, dependent on isoforms [45], and binds with a Kd of ~2–3 μM to apoM [43]. The physiological relevance of apoM as a carrier of retinoic acid is still unknown. Also, apoM binds oxidized phospholipids [28]. Plasma oxidized phospholipids are expected to circulate in the range of 0.1–1 μM in humans. The oxidized phospholipids bind apoM with an IC_50_ ranging from 0.32–0.57 μmol/L [28]. It is interesting to speculate that oxidized phospholipids may displace S1P from apoM during a state of increased oxidative stress [14,28] and, as such, perturb the physiological function of apoM mediated by S1P during diseases with oxidative stress, such as atherosclerosis, but this hypothesis needs further investigation. Interestingly, myristic acid is able to partly displace oxidized phospholipids bound to apoM [28].

### 2.2. ApoM Affects Plasma S1P Levels

To assess the importance of the ability of apoM to bind S1P *in vivo*, plasma S1P was measured in mice that overexpress human apoM 10-fold (*apoM-Tg^H^*) or two-fold (*apoM-Tg^N^*), as compared with the endogenous apoM concentration in wild-type (WT) mice and in apoM-deficient (*apom*^−/−^) mice [19]. S1P was increased by +267% in *apoM-Tg^H^* mice, +71% in *apoM-Tg^N^* and decreased by −46% in *apom*^−/−^ mice compared to WT mice [19]. This effect of apoM on plasma S1P has been confirmed in independent lines of apoM-gene modified mice by Karuna *et al.*[39]. Thus, plasma S1P is affected by the plasma concentration of apoM (see Table 1).

The fraction of plasma S1P that resides in HDL is only present in HDL particles that contain apoM [19]. Hence, S1P is absent in the HDL fraction in *apom*^−/−^ mice [19], as well as in the apoM-free fraction of human plasma HDL [19]. Nevertheless, Karuna *et al.*[39] could not see a statistically significant correlation between plasma apoM and S1P concentration in 51 healthy individuals and 50 patients suffering from disturbances in the HDL metabolism. This may reflect that the fraction of S1P that is bound to albumin contributes more to the variation in total plasma S1P than that bound to apoM-containing HDL. To test this, it would be interesting to study the correlation between apoM and S1P, specifically in the HDL fraction. Alternatively, plasma apoM may not be fully saturated with S1P, in line with the moderate affinity of apoM for S1P. Indeed, Christoffersen *et al.*[19] calculated that the molar ratio between HDL-S1P and plasma apoM was 1:3 in WT and *apoM-Tg^N^* and 1:6 in *apoM-Tg^H^* mice, and similar results were found by Karuna *et al.*[39] in human samples. Moreover, apoM is capable of binding other lipophilic substances, such as oxidized phospholipids [28] and retinol [43], that may compete with S1P binding, thus abrogating a correlation between total plasma apoM and S1P.

### 2.3. ApoM and S1P Affect Lipoprotein Metabolism

When Axler *et al.*[21] established reference intervals for human apoM in normal individuals (*n* = 598), they found that plasma apoM not only correlates with HDL-cholesterol (as 96% of apoM is bound to HDL [14]), but surprisingly also with LDL-cholesterol and total cholesterol. These associations with plasma HDL- and LDL-cholesterol have been confirmed in several other studies [53,54], suggesting a link between cholesterol metabolism and apoM.

In *apoM-Tg^H^* mice, total plasma cholesterol is increased by +13%–22%, whereas it is reduced by −17%–21% in *apom*^−/−^ mice [23] (see Table 1). The mouse, by nature, carries the main part of the plasma cholesterol in HDL, whereas only a minor portion of plasma cholesterol is transported in LDL/VLDL. Indeed, apoM mainly affected plasma HDL-cholesterol when manipulated in a WT mouse [23]. Interestingly, when LDL-receptor-deficient (*ldlr*^−/−^) mice that have elevated LDL/VLDL levels were crossbred with *apoM-Tg^H^* mice, it resulted in a marked 70% increase of total plasma cholesterol, which was due to elevation of plasma LDL/VLDL-cholesterol. In contrast, apoM deficiency in *apom*^−/−^*ldlr*^−/−^ mice resulted in a 25% reduction of plasma LDL/VLDL-cholesterol [25]. Remarkably, apoM can exchange among lipoproteins [25], even though it is anchored to the lipoprotein surface by its hydrophobic signal peptide [55]. Hence, increased numbers of LDL/VLDL particles also increase the relative amount of the total pool of apoM present in LDL/VLDL particles [25,56].

Taken together, the available data thus support the conception that apoM affects the metabolism of LDL. One reason for a VLDL/LDL-cholesterol-increasing effect of apoM could be that clearance of apoM-enriched VLDL/LDL is slower than the clearance of apoM-poor VLDL/LDL [25], an effect that is independent of apoE [25]. However, the mechanism remains obscure at this stage and likely also involves effects of its ligand S1P on plasma lipid metabolism. Indeed, S1P can influence plasma lipids in mice [46,52] (see Table 1). S1P is produced from sphingosine by sphingosine kinases and can be degraded by S1P lyase into phosphoethanolamine and hexadecanal [57]. Mice lacking S1P lyase have four-fold higher serum S1P [46] and significantly increased plasma cholesterol (220 mg/dL *versus* 90 mg/dL) [46]. This suggests that S1P increases plasma cholesterol. The mechanism is unknown. The S1P lyase-deficient mice had increased levels of Sphingomyelin [46]. Sphingomyelin can indirectly affect cholesterol metabolism through the sterol regulatory element-binding proteins (SREBPs) in the endoplasmatic reticulum. Hence, the SREBPs can regulate genes important for cholesterol metabolism [58]. The S1P lyase-deficient mice also had an elevated ceramide level [46]. Ceramide can stimulate cholesterol efflux via ABCA1, which might increase HDL levels [59].

Moreover, treatment of *apoe*^−/−^ mice with a high dose of the S1P analogue FTY720 (3.0 mg/kg/d) resulted in a 2.4-fold increased plasma cholesterol, mainly due to elevated VLDL-cholesterol [52] (see Table 1). A lower dose of FTY720 (0.04–0.4 mg/kg/d) had no effect on plasma cholesterol [50]. Moreover, FTY720 increases the hepatic expression of genes involved in cholesterol and VLDL metabolism, including SREBPs, HMG-CoA reductase, microsomal triglyceride transfer protein and LDL-receptor-related protein-1 (LRP1), indicating that S1P indeed affects lipid metabolism [52].

### 2.4. VLDL/LDL Clearance Affects Plasma ApoM Levels

Studies in mice suggest that not only apoM affects VLDL/LDL metabolism, but perturbations of the plasma LDL/VLDL level can also affect apoM levels. Hence, *ldlr*^−/−^ mice and mice with dysfunctional LRP1 have approximately two-fold increased plasma apoM levels [25]. The increase in plasma apoM was not simply due to elevated plasma cholesterol, since *apoe*^−/−^ mice have unchanged plasma apoM [25]. Interestingly, human individuals with mutations in the LDL-receptor and resulting familial hypercholesterolemia also have increased plasma levels of apoM [56]. Even more remarkable, the fractional catabolic rate of LDL inversely correlates to plasma apoM in healthy humans [56]. Hence, both data from mice and humans support the hypothesis that clearance of LDL can influence plasma levels of apoM.

### 2.5. ApoM Influences Atherosclerosis

Atherosclerosis is a chronic disease of the vessels characterized by cholesterol deposition and the presence and activation of macrophage foam cells and T-lymphocytes [60]. Both apoM and S1P are believed to affect atherogenesis (see Table 1).

The first evidence that apoM overexpression can influence atherosclerosis was provided by Wolfrum *et al.*[24]. Female *ldlr*^−/−^ mice, which were injected with an adenovirus containing the mouse *apom* gene, had ~30% less aortic lesions than *ldlr*^−/−^ control mice [24]. This athero-protective effect of apoM overexpression has been confirmed in *apoM-Tg^N^ldlr*^−/−^ mice with two-fold over-expression of human apoM [23] and in *apoM-Tg^H^apoe*^−/−^ mice with 10-fold overexpression of human apoM [25]. Common for these models, cholesterol levels were only modest or not affected by apoM manipulation [23–25]. The mechanism by which apoM may protect against atherosclerosis may involve effects on lipid metabolism, including increased pre-β HDL formation [23], increased cholesterol efflux from foam cells [14,23] and protection of HDL against oxidation [23,28]. However, as discussed above, apoM has profound effects on both VLDL/LDL metabolism and S1P that also interfere with atherosclerosis, and the net effect of apoM (if any) on cardiovascular disease (CVD) risk in humans is unresolved.

Hence, in a case-control study of CVD affected individuals and controls, there was no association between the plasma apoM concentration and the presence of CVD [53]. Moreover, 10-fold overexpression of human apoM and deficiency of apoM in *ldlr*^−/−^ mice resulted in, respectively, increased and decreased atherosclerosis [25]. In these models, however, apoM also had a marked cholesterol-raising effect that likely counteracted any protective effect of apoM *per se*. In addition, apoM affects S1P metabolism, which could also have vital effects on vascular biology and atherogenesis.

### 2.6. S1P Influences Atherosclerosis

As described above, S1P has many biological effects that involve vascular functions [32,33,40,42], and it is still unresolved if S1P is pro- or anti-atherogenic [61]. A potential pro-atherogenic property of S1P likely includes the ability of S1P to activate the NF-κB pathway [62]. The NF-κB pathway is known among others to activate inflammatory cells, induce cytokine production and promote cell adhesion and, as such, is pro-atherogenic [63]. On the other hand, S1P stimulates eNOS expression through both S1P_r1_[64] and S1P_r3_[42], thereby enhancing the production of endothelial NO and reducing monocyte activation and adhesion, both of which are processes that protect against development of atherosclerosis.

It is unknown which down-stream pathways are important for the effects of plasma S1P on atherosclerosis. Deletion of the S1P_r2_ receptor in *apoe*^−/−^ mice reduces atherosclerosis, suggesting that signaling through S1P_r2_ is pro-atherogenic [48], whereas deletion of the S1P_r3_ receptors did not affect plaque size, but reduced the plaque content of macrophages and smooth muscle cells [49].

Recently, differences in functionality of HDL-bound S1P and albumin-bound S1P have been suggested. Thus, only HDL-bound S1P, but not albumin-bound S1P, activates and sustains high levels of S1Pr1 in endothelial cells [41]. Interestingly, ApoM-S1P-containing HDL, in contrast to apoM-free HDL, binds to the S1P_r1_ and induces formation of tight junctions between human endothelial cells [19]. Hence, apoM-deficient mice with only albumin-bound S1P, and no HDL-bound S1P, display vascular leakage in the lungs [19]. These observations provide an additional mechanism by which apoM could provide anti-atherogenic effects.

Relatively few studies have directly addressed the effect of S1P on atherosclerosis (see Table 1). *Ldlr*^−/−^ mice treated with Sphingosine kinase inhibitors had 30% reduced plasma S1P levels, but no change in plaque size [47]. In further support of a role of S1P in atherosclerosis, FTY720 reduced atherosclerosis in a dose-dependent fashion in *ldlr*^−/−^ mice [50]. Keul *et al.*[51] found a protective effect in cholesterol-fed *apoe*^−/−^ mice, whereas Klingenberg *et al.*[52] observed no effect of FTY720 on atherosclerosis in chow-fed *apoe*^−/−^ mice. In the latter study, FTY720, however, increased plasma cholesterol by 2.4-fold, which may have counteracted the anti-atherogenic effect of FTY720.

Interestingly, patients with coronary artery disease have decreased plasma S1P and HDL-S1P levels compared to controls [37]. In contrast, when plasma S1P levels were adjusted by HDL-cholesterol levels, the S1P/HDL-cholesterol ratio was increased in patients with coronary artery disease. Accordingly, as expected, the HDL-cholesterol level was significantly lower in the coronary artery disease patients [37]. In a second study of patients with ischemic heart disease, serum S1P levels were compared to control subjects with a matched level of HDL-cholesterol [65]. The study confirmed that patients with ischemic heart disease had reduced S1P levels in the VLDL/LDL free serum fraction. Total serum S1P levels were unchanged between controls and patients with ischemic heart disease. Whether apoM and S1P levels in HDL fractions have a potential as diagnostic or prognostic markers in ischemic heart disease needs further investigation.

## 3. Perspectives

Recently, the identification of apoM as a chaperone for S1P has opened a completely new field, which should be explored to better understand the biology of apoM and S1P. S1P, like apoM, has both pro-atherosclerotic and anti-atherosclerotic effects. Thus, it is tempting to speculate that at least some of the biological effects of apoM are mediated by S1P and that some effects of S1P are dependent on apoM. Therefore, it remains to be investigated, for instance, how S1P affects pre-β HDL formation and how apoM affects S1P-dependent processes, such as lymphocyte trafficking. Importantly, FTY720 was recently approved as a treatment option in multiple sclerosis, which is a chronic disease where treatment must be continued for many years. It seems pertinent to explore how FTY720 affects plasma lipids and how this may affect CVD risk in patients with multiple sclerosis.

The effects of apoM and the bioactive S1P (and its five receptors) on lipid metabolism and atherosclerosis are still poorly understood. As indicated above, studies in genetically modified mice and *in vitro* indicate that apoM has several potentially beneficial effects on pre-β HDL formation, oxidation of lipids, cholesterol efflux and atherosclerosis. On the other hand, apoM adversely affects plasma levels of atherogenic lipoproteins that may counteract potential beneficial effects. A better understanding of the biology of apoM appears to be an important foundation towards unraveling the complex biology of HDL. Ultimately, this will enable tailoring HDL-targeting treatments with safe and beneficial effects.

## Figures and Tables

**Table 1:** Effects of apolipoprotein M (apoM)- and sphingosine-1-phosphate (S1P)-modulation on plasma apoM/S1P levels, plasma cholesterol levels and atherosclerosis.

Genotype	ApoM level	S1P level	Cholesterol level	Atherosclerosis
apoM-Tg^H^*[19,23]	++	++	+	Unknown
apoM-Tg^N^*[23]	+	+	0	Unknown
*apom*^−/−^ *[23,39]	−−	−	−	Unknown
*apoM-Tg^H^ldlr*^−/−^ **[25]	++	Unknown	++	+
*apoM-Tg^N^ldlr*^−/−^ **[23,25]	+	Unknown	0	−−
*apom*^−/−^*ldlr*^−/−^ **[25]	−−	Unknown	−	−−
*apoM-Tg^H^apoe*^−/−^ ***[25]	++	Unknown	0	−−
*S1Plyase*^−/−^ *[46]	Unknown	++	++	Unknown
*ldlr*^−/−^+ Sphingosine kinase inhibitor **[47]	Unknown	-	0	0
*apoe*^−/−^*S1P_r2_*^−/−^ ***[48]	Unknown	Unknown	0	−−
*apoe*^−/−^*S1P_r3_*^−/−^ ***[49]	Unknown	Unknown	0	0
*ldlr*^−/−^+ FTY720 (0.04 mg/kg/day) **[50]	Unknown	Unknown	0	0
*ldlr*^−/−^+ FTY720 (0.4 mg/kg/day) **[50]	Unknown	Unknown	0	−
*apoe*^−/−^+ FTY720 (1.25 mg/kg/d) ***[51]	Unknown	Unknown	0	−−
*apoe*^−/−^+ FTY720 (3 mg/kg/d) ***[52]	Unknown	+	++	0

Notes: Overview of the different apoM and S1P mouse models discussed in this review, and the effect on plasma levels of apoM, plasma or serum levels of S1P, plasma or serum levels of cholesterol and on atherosclerotic lesions compared to control mice.

* Effects compared with wild-type mice;

** Effects compared with (untreated) *ldlr*^−/−^ mice;

*** Effects compared with (untreated) *apoe^−/−^* mice. + indicates increase; – indicates decrease; and 0 indicates no change. *ApoM-Tg^H^*, mice with 10-fold increase of human apoM; *apoM-Tg^N^*, mice with two-fold increase of human apoM; *apoM*^−/−^, mice with apoM-deficiency; *ldlr*^−/−^, mice with LDL-receptor deficiency; *apoe*^−/−^, mice with apoE-deficiency; *S1P_r2_*^−/−^, mice with S1P-receptor 2 deficiency; *S1P_r3_*^−/−^, mice with S1P-receptor 3 deficiency; *S1Plyase*^−/−^, mice with S1P lyase deficiency; FTY720, S1P-analogue.
